# Structural factors and best practices in implementing a linkage to HIV care program using the ARTAS model

**DOI:** 10.1186/1472-6963-10-246

**Published:** 2010-08-20

**Authors:** Jason Craw, Lytt Gardner, Amber Rossman, DeAnn Gruber, O'Donnell Noreen, Diana Jordan, Richard Rapp, Cathy Simpson, Karen Phillips

**Affiliations:** 1 Northrop Grumman Corporation, Atlanta, GA, USA; 2 Centers for Disease Control and Prevention, Division of HIV/AIDS Prevention, Atlanta, GA, USA; 3 Kansas City Free Health Clinic, Community Services Department, Kansas City, MO, USA; 4 Louisiana Office of Public Health, HIV/AIDS Program, New Orleans, LA, USA; 5 South Carolina Department of Health and Environmental Control, Columbia, SC, USA; 6 Virginia Department of Health, Division of Disease Prevention, HIV Care Services, Richmond, VA, USA; 7 Center for Interventions, Treatment and Addictions Research, Wright State University Boonshoft School of Medicine, Dayton, OH, USA; 8 University of Alabama at Birmingham, School of Public Health, Department of Health Behavior, Birmingham, AL, USA; 9 Health Services Center, Inc., Anniston, AL, USA

## Abstract

**Background:**

Implementation of linkage to HIV care programs in the U.S. is poorly described in the literature despite the central role of these programs in delivering clients from HIV testing facilities to clinical care sites. Models demonstrating success in linking clients to HIV care from testing locations that do not have co-located medical care are especially needed.

**Methods:**

Data from the Antiretroviral Treatment Access Studies-II project ('ARTAS-II') as well as site visit and project director reports were used to describe structural factors and best practices found in successful linkage to care programs. Successful programs were able to identify recently diagnosed HIV-positive persons and ensure that a high percentage of persons attended an initial HIV primary care provider visit within six months of enrolling in the linkage program.

**Results:**

Eight categories of best practices are described, supplemented by examples from 5 of 10 ARTAS-II sites. These five sites highlighted in the best practices enrolled a total of 352 HIV+ clients and averaged 85% linked to care after six months. The other five grantees enrolled 274 clients and averaged 72% linked to care after six months. Sites with co-located HIV primary medical care services had higher linkage to care rates than non-co-located sites (87% vs. 73%). Five grantees continued linkage to care activities in some capacity after project funding ended.

**Conclusions:**

With the push to expand HIV testing in all U.S. communities, implementation and evaluation of linkage to care programs is needed to maximize the benefits of expanded HIV testing efforts

## Background

Testing, linkage to care, and HIV treatment have been recognized as the three arms of an approach to HIV prevention receiving recognition and funding recently in the U.S. [1]. However, of the three, linkage to care is by far the least well described in the HIV literature. The emergence of several recent publications on the consequences of failure to remain in care [2] and failure to engage in spite of pre-arranged clinic appointments [3,4] has raised the awareness of the problem of sub-optimal rates of early entry into HIV care previously documented [5,6].

Programs that actively facilitate connecting a recently diagnosed HIV-positive person to HIV medical care are known as linkage to care programs or models. Linkage to care models have been mostly homegrown, small single-site efforts at large medical centers closely co-located with HIV testing facilities. Some of these models from large medical centers with emergency room testing and a nearby HIV clinic have shown impressive rates of linkage to care within a few months, often exceeding 85% [7-11]. However, there is still an urgent need to evaluate linkage models that connect clients to HIV care from testing locations outside of HIV medical care settings.

CDC funded a linkage model incorporating multiple sources of clients and testing locations (the Antiretroviral Treatment Access Study [ARTAS-I]); the study used a two-arm randomized controlled trial that compared a brief strengths-based case management intervention with standard-of-care referral in linking recently diagnosed HIV-infected persons to a primary medical care provider [12]. Participants in the intervention group received up to five sessions with a case manager over 90 days. Participants in the standard-of-care (control) group received information about HIV and local care resources, as well as a passive referral to a local HIV medical care provider. Successful initial linkage to care was defined as attending at least one HIV medical care visit within six months after enrollment. The ARTAS-I trial found that 78% in the case management arm versus 60% in the standard-of-care control arm attended at least one HIV primary care visit within six months [12].

Based on positive outcomes from the ARTAS-I trial, CDC funded a demonstration project (ARTAS-II) to evaluate the feasibility of implementing that same linkage to care model in local and state health departments and non-profit community-based organizations (CBOs). Ten health departments or CBOs were funded to implement the linkage to care program. Results from ARTAS-II showed that 79% of all participants enrolled in the program visited an HIV clinician at least once within the first six months [13].

These findings indicated that a brief linkage case management intervention can be implemented effectively by service-oriented organizations in typical HIV programs in rural, mid-sized, and urban settings in the U.S. The objective of the current report was to describe best practices and structural characteristics of organizations from the ARTAS-II demonstration project, specifically those factors which facilitated successful implementation of a program to link recently diagnosed HIV-infected persons to HIV care providers.

## Methods

The best practices, characteristics, and structural factors in this report incorporate information obtained from the 10 ARTAS-II study sites from October 2004 to June 2007 (Table 1). This information was collected from CDC's main data collection activities (as previously reported [13]), from CDC site visit reports, final grantee site reports, and discussions held during the investigator meeting in December 2006. The research team synthesized the information obtained from these sources and created eight categories of best practices in addressing implementation challenges. These practices are elements of program implementation that multiple study personnel considered critical to the overall success of the linkage to care program (Table 2). A ninth category - post-project continuation - describes linkage program continuation efforts. Specific examples illustrating how sites overcame implementation challenges come from project directors at five sites which sustained linkage activities in some capacity for at least several months since project funding ended. These grantees and their corresponding implementing site locations (in parentheses) were: Health Services Center (Anniston, AL); Kansas City Free Health Clinic (Kansas City, MO); Louisiana Office of Public Health, HIV/AIDS Program (Baton Rouge, LA); South Carolina Department of Health and Environmental Control (Columbia & Greenville, SC); and Virginia Department of Health (Richmond, VA).

**Table 1:** Characteristics of ARTAS-II Linkage to Care Study Sites, October 2004 - June 2007

Site/Location	Grantee Agency	Implementing Agency	No. Enrolled	No. (%) Linked^† ^to Care	Co-located* medical care	Post-project^‡ ^continuation
1. Anniston, AL	CBO	CBO	42	39 (92.9%)	Yes	Yes
2. Baton Rouge, LA	State HD	CBOs	72	55 (76.4%)	No	Yes
3. Columbia/Greenville, SC	State HD	CBOs	93	86 (92.5%)	Mixed**	Yes
4. Kansas City, MO	CBO	CBO	89	74 (83.1%)	Yes	Yes
5. Richmond, VA	State HD	CBO	56	45 (80.4%)	No	Yes
**GROUP^¶ ^AVERAGE**			**352**	**299 (84.9%)**		
						
6. Atlanta, GA	CBO	CBO	77	44 (57.1%)	No	No
7. Baltimore, MD	CBO	CBO	22	15 (68.2%)	Yes	No
8. Chicago, IL	CBO	CBO	36	26 (72.2%)	No	No
9. Duval Co./Jacksonville, FL	Local HD	Local HD	64	55 (85.9%)	Yes	No
10. Miami, FL	State HD	Local HD	75	58 (77.3%)	No	No
**GROUP AVERAGE**			**274**	**198 (72.3%)**		
						
**OVERALL**			**626**	**497 (79.4%)**		

ARTAS = Antiretroviral Treatment Access Studies

CBO = community-based (non-profit) organization

HD = health department

^†^Attended at least 1 HIV primary care visit within 6 months of enrolling in program

*HIV primary medical care located on the same campus as the linkage to care program

‡Site was able to continue the linkage to care program in some capacity after CDC funding ended

**One of the two implementing sites in Columbia did not have co-located medical care; the CBO in Greenville had co-located medical care.

^¶^These first five sites provided examples of best practices

**Table 2:** Best Practices in Progam Implementation Identified from the ARTAS-II Linkage to Care Demonstration Project, October 2004 - June 2007

1) Start-up: selecting an appropriate organization to implement a linkage to care program
2) Initiating and implementing: establishing and strengthening essential partnerships for a linkage to care program
3) Distinguishing ARTAS: differentiating linkage case management from long-term case management
4) Marketing the program: communicating the benefits of the linkage to care program to partners
5) Sustaining referrals: successful strategies for obtaining and sustaining referrals to the linkage to care program
6) Transportation for the linkage coordinator: the advantage of being mobile
7) Graduated disengagement: transitioning clients from ARTAS linkage case management to long-term case management
8) Support through supervision: providing consistent and ongoing support and supervision to the linkage coordinators
9) Post-project continuation of linkage programs: the experience of five sites.

ARTAS = Antiretroviral Treatment Access Studies

The study sites implementing ARTAS-II enrolled recently diagnosed HIV-positive participants who were not yet in medical care, with a goal of linking a minimum of 75% of enrolled participants to medical care. The recruitment period for the project was extended from 6 months to 12 months to achieve an overall enrollment of 600 participants.

### The ARTAS Case Management Model

The ARTAS linkage case management model is rooted in strengths-based case management principles. A central strengths-based principle is that clients identify their own strengths, abilities, and assets to overcome barriers and accomplish specific goals. The process of identifying and reinforcing personal strengths enables clients to appreciate their own past self-efficacy, enhances motivation, and prepares them for identifying and achieving goals. Strengths-based case management is designed to help clients identify needed resources to resolve the various individual and structural barriers to HIV medical care [14-16]. The ARTAS case management model also emphasizes smaller client caseloads than traditional case management because of the intensive, short-term nature (up to 5 sessions within 90 days) of the linkage intervention. A summary of the principles of strengths-based case management is listed below.

#### Principles of Strengths-Based Case Management

1. Encourage clients to identify and use their strengths, abilities, and assets to accomplish goals

2. Recognize and support client control over goal-setting and the search for needed resources

3. Establish an effective working relationship with the client

4. View the community as a resource and identify informal sources of support (family members, friends, neighbors, support groups, etc.)

5. Conduct case management as an active, community-based activity (meet with clients in the community, outside of the office setting)

## Results

### Characteristics, Linkage to Care Rates, & Structural Factors of Implementation Sites

Grantee and implementing site characteristics, enrollment totals, and rates of linkage to HIV care are presented in Table 1. Sites numbered 1 through 5 are those which contributed examples of best practices employed to overcome challenges in recruiting and linking participants to medical care. These five sites collectively enrolled 352 participants (56% of the total enrolled) and linked 85% to HIV medical care. The remaining five sites (numbered 6 through 10) enrolled 274 participants (44% of the total enrolled) and linked 72% to HIV medical care. Sites that had co-located HIV medical care on the same campus achieved an 87% linkage to care rate compared with 73% for non-co-located sites.

### Best Practices in Addressing Implementation Challenges (Table 2)

#### 1) Start-up: selecting an appropriate organization to implement a linkage to care program

In the application process, grantees were competitively selected from health departments in the southeastern United States and non-profit CBOs throughout the U.S. Of the 10 funded grantees, there were 4 state health departments, 1 county health department, and 5 CBOs.

Following the funding award, grantees selected an appropriate agency to implement the linkage program. Based on the grantees' final reports, the agencies best suited to implement such a program were health departments (state, county, or city) or CBOs with strong, established working relationships with high-volume HIV testing sites.

##### Linkage to care programs - state health department grantees

For the four state health department grantees, a staff person located in the office of the state AIDS director typically served as the project director. This staffing decision ensured that the project director was familiar with the statewide Ryan White HIV/AIDS Treatment Modernization Act programs and was in a position to select the best agency to implement the linkage program. None of the state health department grantees directly implemented the linkage program; instead, state health departments selected CBOs or local health departments to serve as the implementing organizations. Project directors selected implementing organizations through a non-competitive process, which resulted in the selection of capable and experienced service agencies to implement the linkage program. Collectively, the four state health department grantees enrolled an average of 74 participants compared with non-state health department grantees that enrolled an average of 55 participants (from data in Table 1). A key reason for the enrollment success of the state health department grantees was their level of control in selecting the appropriate organization(s) to implement the linkage program.

Health department project directors considered many factors when selecting community partners to implement the linkage to care program. One important consideration was selecting service organizations with a longstanding history of successful collaboration with the state HIV/AIDS program or organizations that had an existing contractual relationship with the state HIV/AIDS program. Selecting such an agency ensured that staff were familiar working with the state and had a pre-established level of trust and partnership. An equally important factor was selecting an organization that possessed the capacity to deliver or link HIV-infected persons with comprehensive HIV/AIDS services (including HIV counseling and testing, HIV case management, HIV primary medical care, mental health services, substance abuse treatment, housing, and outreach services). Other factors considered when selecting an implementing organization were the organization's level of interest and commitment to offering linkage to care services to HIV-infected clients and their capacity to collect and manage data that would be used to evaluate the linkage program. Lastly, the project directors at state health departments considered selecting organizations in regions of the state that had the highest or increasing rates of new HIV infections. In Louisiana, for example, HIV/AIDS program staff reviewed counseling and testing data to select a region of the state in which to implement the linkage to care program. Once the Baton Rouge region was selected, these data were used to identify potential referring sites (e.g. parish/county health units, STD clinics, and CBOs) that tested a high volume of clients and had high positivity rates.

Other sites considered the ease with which the linkage to care program could be integrated into existing HIV care delivery systems. In South Carolina, for example, the Department of Health chose to implement the linkage program in two regions of the state where there was only one Ryan White Part B provider, which streamlined integration of the linkage program with HIV prevention and care services in those regions.

##### Linkage to care programs - CBO grantees

Five grantees were non-profit CBOs, and in all cases that same CBO served as the implementing agency. Two directly-funded CBO grantees that we highlight in this report are the Kansas City Free Health Clinic and Health Services Center (Alabama). Both these CBOs offered co-located comprehensive HIV/AIDS services, as outlined above. In addition, these two CBOs had pre-existing, strong working relationships with the local health departments serving their community, which facilitated the process of securing cooperation from health department disease intervention specialists (DIS) and counseling and testing staff to supply referrals of recently diagnosed HIV-positive persons. One common tactic used by CBO staff to market the linkage to care program to health departments was to emphasize the reduced burden on health department staff for linkage to care, thus freeing up staff to perform other core job duties, such as partner services.

One of the logistical challenges encountered by CBOs was the lack of office space at local health departments for housing the linkage coordinators (persons delivering the linkage intervention to clients). The CBO in Alabama, for example, addressed this issue by securing an agreement with the local health department to pay monthly rental assistance for onsite office space within the health department to house the linkage coordinators. This onsite office space allowed the linkage coordinators to be nearby to respond quickly to referrals and meet with clients soon after receiving an HIV-positive diagnosis at the health department. In sum, pre-existing relationships with local public health authorities provided a foundation for the goodwill needed for CBOs to secure consistent referrals of recently diagnosed HIV-infected persons.

#### 2) Initiating and implementing: establishing and strengthening essential partnerships for a linkage to care program

One key step in initiating a linkage to care program was for the implementing agency to identify appropriate agencies and organizations in the community with which to partner. These collaborations involved partnering with 1) agencies conducting HIV testing, 2) HIV primary care providers to whom clients could be linked to medical care, and 3) case management agencies to which clients could be referred for long-term case management services after completing the brief linkage to care intervention. One structural attribute that facilitated successful implementation was having as many HIV services as possible (e.g. HIV testing, primary medical care, and long-term case management) co-located within the same building or campus where the linkage case management intervention was being delivered. Sites that had co-located services averaged 87% of participants linked to care, whereas sites without co-located services averaged 73% of participants linked to care (from data in Table 1). Where services were not co-located, active and ongoing efforts to establish and maintain partnerships with HIV testing and care providers were critical.

##### Sources of referrals of HIV-infected persons to the linkage to care program

Many project directors quickly recognized the importance of collaborating closely with DIS staff and counseling and testing staff that had direct access to clients testing HIV-positive. During the first 4 months of recruitment, the CBO in Richmond (VA) enrolled 1 to 2 persons per month into the linkage program. To counteract this period of slow enrollment, the Virginia Department of Health chose to mandate that all DIS staff in the central Virginia region refer all persons testing HIV-positive to the linkage to care program. After the DIS referral mandate was implemented, the CBO averaged over 6 enrollments per month during the next 4 months. The state health department actively monitored DIS referrals to the linkage program and created a mechanism for receiving feedback from the linkage program (a letter mailed back to the DIS) to indicate whether or not the client had been successfully linked to HIV primary care.

In South Carolina, the implementing agency in Greenville had an HIV prevention services employee who essentially functioned as part of the DIS team and had office space in the local health department, so the referral process was relatively seamless. However, due to the large size and complex structure of the health department in Columbia, planning and coordination was required to ensure that DIS and counseling and testing staff were actively referring persons testing HIV-positive to the linkage program. For the first several months of the project, the linkage coordinators and their supervisors met frequently with health department staff to remind them of the new protocol for contacting the linkage coordinators when HIV-positive test results were to be delivered. Once the linkage coordinators established a presence at the clinic, health department staff began consistently referring patients to them.

In Louisiana and Kansas City, linkage program staff initiated contact with all of the counseling and testing sites in the area, met with key staff, presented program information at staff meetings, and distributed brochures, referral forms, referral flow charts, and other relevant information regarding the linkage program. Many of the counseling and testing sites in Baton Rouge were already familiar with the two CBOs implementing the linkage to care program because these CBOs provided long-term HIV/AIDS case management services. The trust and familiarity with these two CBOs increased cooperation between local counseling and testing sites and linkage program staff in Baton Rouge.

In Alabama, Kansas City, and Virginia, there was some initial resistance among DIS staff at the health departments about relinquishing control of the referral to care process. To alleviate DIS concerns about overlapping job duties, the linkage coordinators emphasized that their role was focused on linking clients to HIV medical care, which would relieve DIS of this additional task and allow them to focus on core job duties such as partner elicitation. DIS staff were generally cooperative if they viewed the linkage coordinator's role as complementary and supportive rather than duplicative of their role. Over time, as the linkage coordinators demonstrated their commitment and competency in providing linkage to care services, DIS staff began trusting them with all referrals of recently diagnosed HIV-positive persons.

Local hospitals conducting HIV testing in the emergency department or inpatient units were not a major source of referrals during the CDC demonstration project, but these venues can provide a steady source of referrals to linkage programs. Given CDC's revised recommendations for HIV testing and the expansion of rapid testing, emergency departments will likely become a more important source of HIV-positive diagnoses [17]. Since the conclusion of the demonstration project, South Carolina has been funded under a CDC expanded testing initiative in emergency departments to implement routine HIV rapid testing in three hospitals. These hospitals are using linkage coordinators to connect patients who are diagnosed HIV-positive in the emergency department with HIV medical care. The linkage program in Kansas City partnered with a local hospital that was initiating "opt-out" testing in their emergency department. Emergency department staff wanted a reliable referral mechanism for persons testing HIV-positive at any time of day, so the Kansas City linkage program provided staff with a 24/7 referral line/pager to directly refer clients to the linkage program. Emergency department HIV testing has become a steady source of referrals to the linkage program in Kansas City since the end of the demonstration project.

##### Partnerships with HIV primary medical care providers

Compared with sites *without *co-located HIV primary medical care and ARTAS case management, persons enrolled at sites *with *co-located services had significantly higher rates of linkage to care at six months in a multivariate analysis [13]. More importantly, however, several implementing sites that lacked co-located HIV medical care (Baton Rouge, Richmond, and one CBO in Columbia, SC) still linked 80% to medical care. Several additional efforts were made by these sites to compensate for not having HIV primary medical care onsite. First, it was essential that linkage coordinators were familiar with local HIV primary care providers in the community to provide accurate information to clients about HIV primary care options. In some instances, these relationships with primary care providers were already firmly established; in others, the linkage coordinators had to establish a relationship with these medical providers and familiarize them with the purpose of the linkage program. Another important factor in successfully linking clients to care was arranging transportation and, in certain instances, accompanying clients to their first medical visit to alleviate potential anxiety or fear about the visit. The flexibility to accompany clients to medical appointments facilitated successful linkage in the absence of co-located HIV primary care.

##### Partnerships with case management agencies

Establishing working relationships and partnerships with local case management agencies enhanced the overall delivery and continuity of client services. At the majority of sites, the agency implementing the linkage program also provided Ryan White-funded case management services. Establishing the linkage program within an agency that provided Ryan White services allowed for a seamless transition for those clients who needed long-term case management upon discharge from short-term ARTAS case management. Regardless of whether or not the long-term case managers were located in-house, it was common for linkage staff to demonstrate how the program benefited case managers, especially emphasizing the preparatory work done by linkage staff (e.g., completing eligibility assessments, intake paperwork) prior to the client's first meeting with the case manager.

#### 3) Distinguishing ARTAS: differentiating linkage case management from long-term case management

From an early stage of implementation, several project directors recognized that a change in job title was needed to reduce confusion and differentiate the roles and responsibilities of the short-term ARTAS linkage case manager from long-term or Ryan White case managers. Nearly all sites dropped the term "case manager" in favor of the term "linkage coordinator" or "linkage to care coordinator" to clarify that this position was transitional, short-term, and specific to facilitating linkage to medical care. Both clients and long-term case managers benefited from this distinction in terminology. In addition to making the linkage coordinator's role more transparent, another benefit of changing the job title was that it helped alleviate fears that the linkage program was competing for or "stealing" potential clients from long-term case managers. Dropping the term "case manager" from the job title helped mitigate territoriality or turf issues that arose when linkage coordinators were perceived as duplicating existing services rather than coordinating clients' entry into HIV medical care. Despite the clarification in job title, however, some Ryan White case managers expressed resentment over the discrepancy in caseload size (at agencies that housed both Ryan White case managers and linkage coordinators). To alleviate these concerns, supervisory staff scheduled periodic educational sessions with the case managers to ensure they had a thorough understanding of the linkage program and explain that smaller caseloads were needed due to the short-term, intensive nature of the intervention.

Many ARTAS-II sites used similar methods to educate partners about the purpose and objectives of the linkage program and to delineate the roles and responsibilities of the linkage coordinator. Several sites held "lunch and learn" meetings to present information about the linkage to care program. Project directors at those sites noted that providing free lunch at these in-service sessions helped encourage attendance. Another common delivery method involved making special presentations to community partners, HIV consortia, and regional case management forums to educate stakeholders about the linkage to care program. Most sites created brochures and other informational materials about the linkage program to disseminate through meetings, in-services, and follow-up mailings. In Virginia, project staff recognized that their initial marketing materials were not effective because they were lengthy and convoluted, so they were replaced with simple, concise pocket cards or flyers that clearly outlined the benefits of the linkage program, which clients to refer, and who to contact. Virginia also created and presented case-based examples of the benefits of using a strengths-based approach to link clients to HIV medical care.

#### 4) Marketing the program: communicating the benefits of the linkage to care program

Program staff consistently emphasized the benefits of the linkage to care program when communicating with potential referring partners and long-term case managers. As was mentioned earlier, linkage coordinators frequently completed intake assessments or eligibility paperwork, which reduced the overall workload for long-term case managers when clients were transitioned to them. Long-term case managers liked receiving clients who had eligibility and intake documentation completed because it freed them to do other work with clients. Another benefit of the program was that ARTAS clients were often better prepared than non-ARTAS clients when transitioned to long-term case management. Several project directors mentioned that the linkage coordinators took time to orient ARTAS clients to the case management system and the services that their case manager could provide. Lastly, the South Carolina sites marketed the linkage program by emphasizing the benefit to the patient's health and the overall public health in that patients who are in regular HIV medical care generally have better health (e.g. lower viral loads) and are less likely to transmit HIV infection than those who are not in HIV care [18,19].

#### 5) Sustaining referrals: successful strategies for obtaining and sustaining referrals to the linkage to care program

Across sites there was fairly uniform agreement about strategies that were most successful in generating and sustaining referrals to the linkage program. Maintaining ongoing communication and frequent contact (e.g. weekly) with supervisory and front-line staff at partner agencies was extremely important in ensuring the success of the referral process. In Virginia, program staff documented and monitored the number and types of contacts initiated by the linkage coordinators to establish new referral sources and partnerships. Documenting and tracking the number, frequency, and outcome of these contacts contributed to an increase in referrals throughout the project period. The Baton Rouge CBOs that implemented the linkage program hosted a quarterly networking lunch with their referring partners to provide updates on the progress of the linkage program and discuss successes and challenges with the referral process. This meeting also provided a forum in which community agencies could connect with each other and learn about the resources and services offered by each agency. Frequent communication with referral partners was crucial not only from a relationship-building perspective but also from a quality improvement perspective. Part of establishing and sustaining consistent referrals to the linkage program involved detecting problems or barriers with the existing referral process in order to make adjustments and improvements to the process. One of the most common issues that caused declines or total stoppage of referrals to the linkage program was staff turnover at referring agencies. When staff turnover occurred, the linkage coordinator had to work with the referral partner to identify a new person who would be responsible for making referrals to the linkage program and re-educate staff on the referral process.

Another key strategy for sustaining referrals was providing referral partners with timely feedback on whether or not the clients they had referred had been linked to care. Providing referral partners with regular feedback was important in establishing trust and confidence in the linkage program and sustaining productive working relationships. In Virginia, DIS staff required formal documentation, such as a letter to place in their files, to confirm whether or not the client had been referred and linked to care. Less formal feedback mechanisms (e.g., phone calls, e-mails, faxes) were also used with referral partners to inform them about the client's linkage status. In Kansas City, linkage staff prepared and disseminated a quarterly newsletter that shared linkage "success stories", praised partners for successful referrals, and provided tips on making referrals to the linkage program. Referral partners were viewed as secondary clients, and providing feedback on referred clients was seen as good customer service. The feedback helped ensure that persons who made referrals received credit for their important role in connecting clients with HIV care.

In addition to ongoing communication and providing referral partners with timely feedback, linkage coordinators also emphasized the importance of responding to all referrals in a timely and consistent manner. Timeliness of response to referrals was often facilitated by the proximity of the linkage coordinator to the referral source. At half of the program sites, the linkage coordinator was located in the same facility where HIV testing was conducted, or was available "on-call" by pager or cell phone to meet with the client in person immediately after the HIV-positive test result was delivered. On-call availability was highly valued by DIS staff in Alabama and Kansas City because the linkage coordinators could be contacted directly and they responded quickly, even assisting the client afterhours if needed. When it was not logistically feasible to meet with a client in person, the linkage coordinator attempted to call while the client was still with the HIV post-test counselor. The phone call provided an opportunity for the linkage coordinator to speak directly with the client, establish some initial rapport, and schedule a convenient time and place to meet with the client to discuss the linkage to care program. This initial, timely phone contact helped ensure that the client would eventually follow up and meet with the linkage coordinator. In Alabama and Kansas City, the linkage coordinators were trained and certified to conduct HIV testing, deliver test results, and provide pre- and post-test counseling. These sites found that coupling HIV counseling and testing with the primary duties of the linkage coordinator was an efficient use of staff time and resources and decreased the likelihood of client referrals falling through the cracks.

#### 6) Transportation for the linkage coordinator: the advantage of being mobile

One of the guiding strengths-based principles is making case management an active, community-based activity. Accordingly, many study sites allowed their linkage coordinators to be mobile and meet with referral partners and clients outside of the office setting (e.g., client's home, coffee shop, library). However, agency-specific policies prohibited some study sites from allowing their linkage coordinators to meet with clients outside of the office or transport clients in a personal or agency-owned vehicle. Nonetheless, the agencies that were allowed to do so found that the flexibility to meet with clients outside of the office setting was critical to the success of the linkage program. Meeting face-to-face with clients immediately after post-test counseling was extremely valuable to the linkage coordinator in building trust and establishing rapport with the client. At some sites, the linkage coordinator was able to use an agency-owned vehicle to meet with clients or referral partners outside of the office. At other sites, the linkage coordinator used a personal vehicle and was reimbursed for mileage. The linkage coordinators were usually covered under the agency's liability insurance for work conducted with clients outside of the office.

A few sites even allowed their linkage coordinators to ride the bus or subway with clients to help them navigate the transportation system. Transportation is a common barrier to engaging in ongoing HIV medical care, and linkage coordinators used the strengths-based approach to help clients learn how to secure transportation and navigate their way to the clinic so they could become self-sufficient in arranging transportation in the future. The majority of sites also encouraged the linkage coordinator to meet clients at the HIV clinic and accompany them during their first HIV medical care visit. Allowing the linkage coordinators to be mobile and meet with clients outside of the office was vital to the success of the linkage program, especially among sites serving large, rural geographic areas where clients frequently had limited access to personal or public transportation.

#### 7) Transitioning clients from ARTAS linkage case management to long-term case management

The short-term nature of the ARTAS linkage intervention requires a seamless process by which clients can transition from ARTAS case management to long-term case management, if needed--a process referred to as disengagement. Several techniques for handling the disengagement process were presented during the intervention training. The linkage coordinators were taught that disengagement is a graduated process that begins during the very first contact with the client and one that is reinforced during subsequent contacts. The client should be informed up front about the short-term, transitional nature and goals of ARTAS linkage case management. The linkage coordinator must ensure that clients fully understand that once they have completed five sessions together or have entered HIV primary care (whichever occurs first), they will be transferred to long-term case management services, if needed.

##### The "active hand-off" model for facilitating the transition to long-term case management

The "active hand-off" is one model for facilitating the client's transition to long-term case management services provided through an HIV medical clinic or community agency. The active hand-off often involved introducing the client to his/her new case manager prior to disengagement and describing the types of case management services offered. Oftentimes, the linkage coordinator attended part of the client's first session with the new case manager. In Kansas City, the linkage coordinators used a statewide case management data capture system, so the comprehensive progress notes and any completed eligibility assessments were readily available to long-term case managers. Some linkage coordinators allowed clients to make a brief phone call after disengagement just to check in and let them know how things were going with their new case manager. The active hand-off model was especially useful when the linkage coordinator and long-term case managers were located at the same agency. Most importantly, clients had the opportunity to meet their long-term case manager prior to disengaging from the linkage coordinator. In addition, because of the intervention sessions with the linkage coordinator, most clients were already familiar with agency staff, agency culture and policies, and how to arrange transportation to the agency.

#### 8) Support through supervision: providing consistent and ongoing support and supervision to the linkage coordinators

One practice shared by the most successful implementing agencies was having a consistent, ongoing supervisory presence for the linkage coordinators. Ongoing supervision helps monitor the fidelity with which the linkage intervention is being delivered and ensures that the linkage coordinators adhere to strengths-based principles in their encounters with clients. Supervision was also critical for monitoring the disengagement process and supporting the linkage coordinators with the more difficult client cases. For all these reasons, it is important that the person supervising the linkage coordinators receive training in strengths-based methodology. The supervisor typically had a background in case management or social work and extensive experience with HIV service delivery systems. At a few sites, this person was responsible for supervising the linkage coordinators and the Ryan White case managers. The most common model of supervision was holding weekly case conference meetings during which client cases were discussed.

Another level of supervision is needed when the state health department is overseeing implementation of the linkage program by community agencies. At this level, a staff person from the state HIV/AIDS program office needs to monitor implementation of the linkage program locally to ensure that the contracted agencies follow protocols and deliver linkage to care services as intended. For example, the linkage program directors at the state health departments in Louisiana, Virginia, and South Carolina monitored the number of referrals to the linkage program from various sources, gave quality assurance oversight, and provided programmatic or administrative support.

#### 9) Post-project continuation of linkage programs

As expected, when CDC funding ceased, 5 of the 10 study sites were unable to continue linkage to care activities. The five sites highlighted in this report fared better, with all five extending linkage activities in some capacity for at least several months beyond the end of project funding. However, these extensions were themselves dependent on small amounts of local funding which, in most cases, could not be sustained in the long run. South Carolina ceased all linkage activities when project funding ended, but re-started them several months later by incorporating linkage to care services in their CDC-funded Expanded HIV Testing Initiative project in emergency departments and in a Ryan White clinic using Minority AIDS Initiative funding. The effort to continue providing linkage services in South Carolina was successful because it used multiple funding streams (CDC, Ryan White Part B, and Minority AIDS Initiative) and employed one of the linkage coordinators from the ARTAS demonstration project. The Alabama CBO also used multiple funding sources to continue providing linkage services using one of the original linkage coordinators as a dual linkage/standard case manager. Louisiana was able to maintain one of the two linkage coordinators on a part-time basis for several months after the end of the project. The state HIV/AIDS programs in Missouri and Virginia were able to use Ryan White funding to continue supporting linkage program activities. Missouri also sponsored training opportunities in strengths-based methodology for case managers throughout the state.

One key to setting the stage for future funding in Missouri was that the linkage program coordinator from Kansas City served on Missouri state committees for case management standards and quality improvement. She took advantage of any opportunity to report linkage to care program data, milestones, case studies, and anecdotal successes to the committee and to the state AIDS Director. In mid-2008, funding was expanded to quadruple the number of clients served by the linkage program in three areas of Missouri (Kansas City, St. Louis, and Springfield/Joplin), which represent very different client populations across urban, suburban, and rural communities. The Kansas City, MO area has expanded the number of linkage coordinators, and has also done consulting work to expand the linkage program to the state of Kansas.

## Discussion

Linkage to care is the important bridge between HIV counseling and testing and HIV medical care. Successful implementation of a linkage to care program requires attention to all the program practices described. As a group, sites that followed these best practices enrolled more participants, linked a higher percentage to medical care, and were able to continue linkage program activities for some period of time after CDC funding ended. It is noteworthy that the five grantees that continued the linkage program came from smaller metropolitan areas (average 990,000 persons in 2000) compared to the five grantees that did not continue (average 4.1 million persons in 2000).

Attention to these eight practices takes on additional urgency when HIV testing is not co-located with an onsite HIV clinic because inter-agency partnerships must be maintained. Strong inter-agency partnerships were a defining characteristic of sites that continued the linkage program after CDC funding ended. When multiple sources of newly diagnosed clients (across agencies) are involved, the organizational complexity to maintain these sources can be daunting. Nevertheless, implementing these best practices is not beyond the ability of even a small CBO. The five CBOs that were able to continue the linkage program after project funding ended had long-standing, cooperative relationships with local health departments, resulting in a steady influx of recently diagnosed HIV-positive persons. These CBOs recruited 56% of the 626 participants and recruited an average of 70 participants per grantee. In contrast, the three CBOs that did not continue the linkage program all experienced major challenges establishing and maintaining reliable relationships with local health departments. These CBOs recruited an average of 45 participants per grantee and averaged less than 70% linked to care.

The strongest performing grantees were often, but not exclusively, those with co-located HIV medical care. As a group, co-located sites averaged 87% linkage to care compared to 73% for sites not co-located. Co-location of services makes initial entry to care easier for most clients. Yet there are many communities in the U.S. where co-location of an HIV clinic with one or more HIV testing sites is neither feasible nor sensible. The experience of these 10 implementing sites showed that even in communities where the linkage program was not co-located with an HIV clinic, achieving a linkage rate of 80% or higher was possible.

Our report is not without limitations. We did not present statistical testing of the differences in linkage to care rates for site-level characteristics and best practices. Site-level best practices tend to be clustered, making separation of these characteristics on a small sample difficult. Modeling of these data that accounts for collinearity of site level factors and includes individual level factors was beyond the scope of this report.

## Conclusions

With the continuing push to expand offering of HIV testing in the United States, there is an urgent need to adapt linkage to care programs so that public health departments, community-based organizations, hospitals, emergency rooms, private doctors' offices, and HIV clinics are affiliated with a linkage to care program. To fully maximize the benefits of expanded HIV testing will require careful implementation, adaptation, and evaluation of linkage to care programs.

## Competing interests

The authors declare that they have no competing interests.

## Authors' contributions

JC was the study coordinator and participated in drafting the manuscript. LG conceived the study and participated in drafting the manuscript. AR managed the Kansas City site of the project and participated in editing the manuscript. DG managed the Baton Rouge site of the project and participated in editing the manuscript. NO managed the South Carolina site of the project and participated in editing the manuscript. DJ managed the Virginia site of the project and participated in editing the manuscript. RR provided the training for the linkage coordinators and participated in editing the manuscript. CS provided guidance to the design and implementation for the Alabama site of the project. KP managed the Alabama site of the project and participated in editing the manuscript.

All authors read and approved the final manuscript.

## Pre-publication history

The pre-publication history for this paper can be accessed here:

http://www.biomedcentral.com/1472-6963/10/246/prepub
